# Pain, Culture and Pedagogy: A Preliminary Investigation of Attitudes Towards “Reasonable” Pain Tolerance in the Grassroots Reproduction of a Culture of Risk

**DOI:** 10.1177/0033294120988096

**Published:** 2021-02-11

**Authors:** Paul K. Miller, Sophie Van Der Zee, David Elliott

**Affiliations:** Institute of Health, 9480 University of Cumbria, Lancaster, UK; Erasmus School of Economics, 6984 Erasmus University Rotterdam, Rotterdam, the Netherlands; Institute of Health, 9480 University of Cumbria, Lancaster, UK

**Keywords:** Attitudes, culture, pain, pedagogy, social psychology, sport

## Abstract

In recent years a considerable body of psychological research has explored the relationship between membership of socio-cultural groups and personal pain perception. Rather less systematic attention has, however, been accorded to how such group membership(s) might influence individual attitudes towards the pain of others. In this paper, immersion in the culture of competitive sport, widely regarded as being exaggeratedly tolerant of risky behaviours around pain, is taken as a case-in-point with students of Physical Education (PE) in tertiary education as the key focus. PE students are highly-immersed in competitive sporting culture both academically and (typically) practically, and also represent a key nexus of cross-generational transmission regarding the norms of sport itself. Their attitudes towards the pain that others should reasonably tolerate during a range of activities, sporting and otherwise, were evaluated through a direct comparison with those of peers much less immersed in competitive sporting culture. In total, N=301 (144 PE, 157 non-PE) undergraduate students in the UK responded to a vignette-based survey. Therein, all participants were required to rate the pain (on a standard 0-10 scale) at which a standardised “other” should desist engagement with a set of five defined sporting and non-sporting tasks, each with weak and strong task severities. Results indicated that PE students were significantly more likely to expect others to persevere through higher levels of pain than their non-PE peers, but only during the sport-related tasks – an effect further magnified when task severity was high. In other tasks, there was no significant difference between groups, or valence of the effect was actually reversed. It is argued that the findings underscore some extant knowledge about the relationship between acculturated attitudes to pain, while also having practical implications for understanding sport-based pedagogy, and its potentially problematic role in the ongoing reproduction of a “culture of risk.”

## Introduction

Pain remains something of an enigma in contemporary scientific investigation. A broad range of research has comprehensively demonstrated that the experience of pain cannot be exclusively explained by the nociceptive system directly responding to noxious stimuli (Bendelow, 2006; Bendelow & Williams, 1995; Garland, 2012). Individuals routinely report pain, sometimes severe and lasting in form, which cannot be accounted for physiologically; a diagnosis of Chronic Pain Syndrome evidences exactly this (Crue & Pinsky, 2009). Others sustain injuries or illnesses that, theoretically at least, should cause significant suffering and yet report little or no discomfort (Winance, 2006). It is now generally, thus, accepted that pain is an “ensemble act” (Miller & Newton, 2006, p. 148) at the juncture of various physiological, psychological and socio-cultural influences (Bendelow & Williams, 1995; Forsythe et al., 2011).

The impact and intersection of psychological and socio-cultural influences has, to date, been extensively demonstrated in how attitudes towards personal pain are reproduced and perpetuated within given groups (Cleland et al., 2005; Edwards & Fillingim, 2001; Forsythe et al., 2011; Wandner et al., 2012). Rather less overall attention has, however, been accorded to how such acculturation informs attitudes towards the pain of others (Coll et al., 2012; Craig et al., 2010; Wandner et al., 2012). Given this, the particular focus of this paper falls upon the relationship between individuals’ immersion in the culture of competitive sport, widely viewed as being exaggeratedly tolerant of physically risky pain behaviours (Curry & Strauss, 1994; Nixon, 1992; Schneider et al., 2019; R. T. Smith, 2008), and those individuals’ attitudes to how others should deal with pain. In short, it is quantitatively explored whether persons who are highly immersed in a culture which is often thought to accept (and even promote) a “no pain, no gain” ethos (Heil, 2012; Nemeth et al., 2005) will come to expect that others should tolerate more pain within given activities (sporting and otherwise) than those who are less so immersed.

### Culture, pain and competitive sport

While some studies have proposed that extensive involvement in physically-demanding sporting activity can increase physical pain threshold (see Spector et al., 1996), a more sustained focus in pertinent social scientific research rests upon how individuals learn/choose to perceive pain and associated physical risks in competitive sporting contexts as acceptable – i.e. to “shrug them off” – in a way that would likely be deemed unwise or unnecessary elsewhere (Madrigal et al., 2015; Tesarz et al., 2012; Wiese‐Bjornstal, 2010). Weinberg et al. (2013) and Saragiotto et al. (2014), for example, have robustly demonstrated that individuals with strong senses of athletic identity tend to exhibit significantly stronger positive attitudes towards playing through high levels of pain than those without, a phenomenon explained by the latter as a consequence of inherently competitive personalities. Safai (2003), meanwhile, more explicitly emphasises context when observing that student athletes are much more likely to tolerate pain, and push to play when hurt, if the game itself is of particular strategic importance to the team (i.e. the collective rewards are more substantial than usual); this being particularly so towards the end of a season. A higher readiness to endure pain (and a lower perception of it) has also been reported when athletes understand that they are being watched by coaches, peers or significant others that they wish to impress, and/or for whom they wish represent “strength” or hide “weakness” (Howe, 2004; Pike, 2005; Pike & Maguire, 2003; Weinberg et al., 2013). Such empirical outcomes remain evocative of Beecher’s (1959) seminal observations emergent of battlefield medicine; individuals can be recurrently shown to perceive less pain, and/or be prepared to tolerate more pain, when highly engaged in contexts that they deem of significance, due to high personal value placed upon the activities therein, or the projected outputs thereof. As a corollary, Jackson et al. (2002) – echoing Bandura’s (1997) classic work on self-efficacy – argue that when an individual pursues a goal that they believe they *should* attain, be that as an outcome of direct prior experience or general social learning, significantly less pain is reported than might otherwise have been expected. Equally, when goal-attainment is perceived to be desirable in terms of enhancing self-image, or its non-attainment is a threat to self, significantly less pain is reported (Jessiman-Perreault & Godley, 2016).

In terms of the active reproduction of attitudes to pain, meanwhile, the manner in which individuals perceive, tolerate and communicate their own pain can – no doubt – influence others through example. “Microscopic” cultural formations such as family membership, for example, are known to be a powerful influence on how children learn to evaluate the relevance or significance of a painful experience, and also how to cope with and communicate it, with parental reaction to particular incidences being a prime determinant (Hechler et al., 2011; Palermo et al., 2014). A range of studies has similarly illustrated that the manner in which individuals handle pain can have profound impacts on the ways in which self-identified peers and/or protégés can then orient to their own (Craig et al., 2010; Weinberg et al., 2013). Within particular cultural groupings, pedagogical agents such as teachers, mentors and coaches can play a pivotal role in the transmission of a range of pain-related attitudes from one generation to the next (Heil, 2012; Howe, 2004; Schneider et al., 2019). This is done not only through their simple provision of a personal example, but through direct inculcation, and through the mobilisation of resources (such as the very right to participate in given activities) which tacitly or explicitly reward certain attitudes towards pain, and punish others (Nixon, 1993; Walk, 1997). For example, in an influential study of a wide variety of sporting organisations, Nixon (1992) reports that even embedded medical personnel are often complicit in exhorting and encouraging athletes to play with pain or injuries. In this study, the pain athletes experienced, which elsewhere might be considered a major cause for concern, was widely viewed by the sport medics as a necessary evil in the quest for sporting success (see also Pike, 2005; Roderick et al., 2000; Safai, 2003). This, in turn, provided a yardstick for athletes in terms of understanding what was and what was not “necessary” pain to contextually endure.

It is bordering upon axiomatic, thus, that attitudes towards personal pain can be strongly shaped through the direct and indirect transmission of attitudinal norms within socio-cultural groups. However, and as noted above, there is less abundant research addressing how this order of factor might govern attitudes towards the pain experienced by others. In papers that do address this issue, it has been demonstrated that caregivers’ own experiences of pain (particularly personal, long-term exposure) can attenuate their ratings of the likely pain being experienced by other people (Coll et al., 2012). There are also findings which indicate that individuals may invoke judgmental heuristics – which are themselves linked to culture and socialised stereotypes (Miller et al., 2012) – when evaluating pain in others (Kappesser et al., 2006). For example, Martel et al. (2011) explore how gendered stereotypes might (variably) impact upon such situated assessments, while Trawalter et al. (2012) highlight how many individuals, including healthcare professionals, often judge that persons from more “privileged” backgrounds likely experience greater pain in response to a given stimulus than those who might have endured more personal hardship in the past. It is the body of work on sports medicine, however, that possibly remains the most compelling case-in-point regarding the topic of this paper, in indicating that physicians themselves who are highly immersed in the culture of sport often take a more laissez-faire approach to the pain of their charges than is typically the case in general medicine (Pike, 2005; Roderick et al., 2000; Safai, 2003).

### Pain, (physical) education and cultural transmission

Given the above, there is evidence from which to infer that immersion in sporting culture to some extent governs attitudes towards pain in others. To date, and outside of the professional/clinical domain, however, this corpus remains largely dominated by qualitative and/or sociologically-leaning investigation (Jessiman-Perreault & Godley, 2016; Kortesluoma & Nikkonen, 2004; Nixon, 1996; Schneider et al., 2019). The stated enterprise herein is to explore the manners in which the cultural factors that might inform personal perceptions of pain can also influence attitudes to how others should tolerate pain across a variety of contexts. It is in this respect that physical educators – and students of physical education (henceforth PE) in particular – provide a valuable case study in terms of wider cultural immersion and, and more mundane (i.e. day-to-day) attitudes to pain.

PE students are likely to be highly immersed in the broader culture of competitive sport, both ideologically and practically (Spittle et al., 2009). Students of PE, particularly at the undergraduate (tertiary education) stage, represent a nexus point between how sports (and other physical activities) are taught, and how they *will be* taught. Furthermore, as prospective teachers, these students are also likely to have direct governance over their charges’ rights within sports and other physical activities in the future, with direct implications for the wellbeing of those charges. By comparing attitudes towards reasonable pain tolerance in others among PE students to attitudes of peers who are not so immersed in competitive sporting culture, the following hypotheses can be evaluated: (1) there will be a greater expectation of pain tolerance for high (competitive) task severities regardless of sporting immersion; (2) immersion in sporting culture will lead to greater expectation of pain tolerance in others in sporting contexts and (3) greater levels of immersion in sporting culture will lead to stronger levels of discrimination between low and high task severities in sporting contexts.

## Methods

### Participants

With full institutional ethical approval, a total of 301 participants were recruited for this investigation; 145 (48%) males and 156 (52%) females; age ranged from 18 to 57 years, (M_age_ = 24.85; SD = 8.28). All participants were undergraduate students at UK universities at the time of data collection. Participants studied PE (N = 144), or subjects unrelated to sports (N = 157; a *de facto* control group). The students who did not study PE were enrolled in the following subjects: Caring sciences (e.g., nursing, medicine and social work; N = 81), education (N = 26), environmental or physical sciences (N = 13), social sciences (N = 32) and other (N = 5). In the present study, 98.6% of PE students actively competed in vigorous competitive sports at least once per week on average, compared to 41.4% of students studying other disciplines.

### Instruments

In a manner conversant with comparable pain research (Bryce et al., 2012; Lafond et al., 2015; Miceli & Katz, 2009), the present study utilised a vignette instrument to assess attitudes towards pain in others. Before moving to address the vignette aspect of the survey, participants were asked to provide key demographic details and their course of study, after which they were presented with the following standardised scenario:

*“Let’s say you have a healthy male friend in his early twenties, who is also a university student. He enjoys socializing and keeping fit, but he is also prone to push himself too hard through pain in a range of activities, and often needs to be told when to stop before he damages himself. You both agree that, in the future, he will tell you how much pain he is in at any time by indicating on a scale between 1 (very little pain) and 10 (the worst pain possible), and if you think that’s too much pain to continue with what he is doing, he’ll stop.”*

They were then provided with ten activities in which to indicate the pain level, on a single-item 1-10 pain scale (Jensen & Karoly, 2001) at which they would they would tell their friend to stop if he began to experience a generalised stomach pain (henceforth “Acceptable Pain Endurance”). The ten activities (shown in Table 1) comprised five vignette-pertinent sporting and non-sporting activities, manipulated for “weak” and “strong” task severity. The vignettes were trialled as a class-based questionnaire and a focus group schedule^1^, to ensure they were study-valid.

**Table 1.** Activities described in survey.

Activity #	“Weak” task severity	“Strong” task severity
A1	A1W: Writing an essay that is due for submission in two weeks.	A1S: Writing an essay that is due for submission the next morning.
A2	A2W: Socialising on a normal Friday night.	A2S: Socialising on a close friend’s birthday.
A3	A3W: Running to keep fit.	A3S: Running in a competitive race.
A4	A4W: Playing “kick-around” football with friends in the park.	A4S: Playing football for a club team in a cup game.
A5	A5W: Taking part in a normal university class.	A5S: Sitting a university exam.

### Procedure

Data were collected exclusively within the UK, using the JISC Online Surveys system. To recruit participants, programme leaders (PE and otherwise, in equal balance) from a number of academic institutions were contacted via email and asked if they would be willing to disseminate the survey to their students. An email link to the survey was subsequently sent to the point of contact who then forwarded to potential participants; there were no constraints on eligibility to participate. Full Informed Consent procedures were included in the survey. The survey remained open for one full month and was then closed.

## Results

### Weak vs. strong tasks

**Figure 1.**
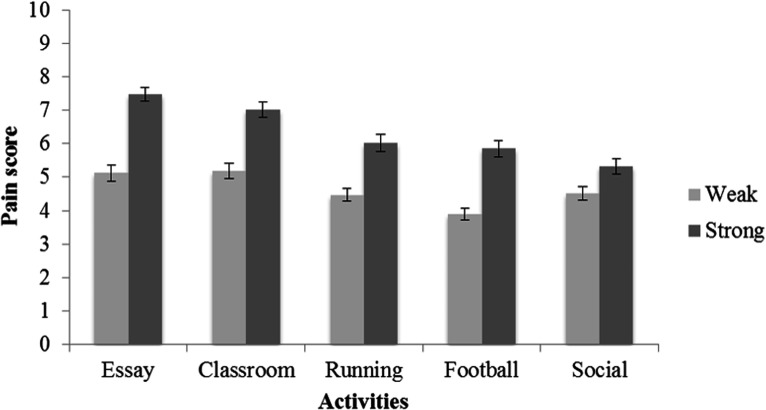
Mean scores for acceptable pain endurance scores during weak and strong activities. Error bars represent 95% confidence intervals.

Firstly, the impact of task severity on acceptable pain endurance scores was examined. A two-way repeated measures ANOVA, with the five Activities and two Task Severity levels (Weak/Strong) as the repeated measures, was performed. Mauchly’s test indicated that the assumption of sphericity was violated for both Activity, *Χ*^2^ (9) = 727.66, *p* < .001, and the interaction between Activity and Task Severity, *Χ*^2^ (9) = 107.05, *p* < .001; as such, the Greenhouse-Geisser correction was applied (for Activity, ε = .43; for the interaction between Activity and Task Severity, ε = .86). The results demonstrated that, overall, participants scored acceptable pain endurance more highly when task severity was strong (*M* = 6.34 ± 1.26) as opposed to weak (*M* = 4.64 ± 1.02; *F*(1, 300) = 790.56, *p* < .001, $\eta_{p}^{2}$ = .73). Additional main effects for Activity, *F*(1.74, 520.41) = 54.91, *p* < .001, $\eta_{p}^{2}$ = .16, and interaction of Task Severity on Activity, *F*(3.42, 1026.29) = 38.17, *p* < .001, $\eta_{p}^{2}$ = .11 were found; see Figure 1 for the mean scores and 95% confidence intervals of all activities. Bonferroni corrected post-hoc analyses were conducted for all 5 activities. The results revealed that all activities significantly differed from each other, apart from the footballing and social activities.

### Immersion in sporting culture

**Figure 2.**
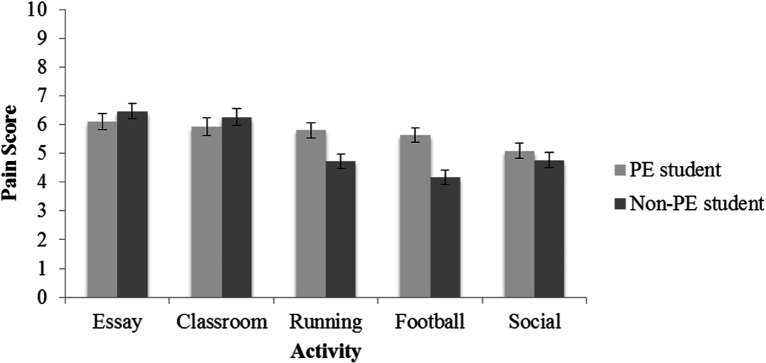
Mean scores for acceptable contextual pain endurance among PE students and non-PE students in different activities. Error bars represent 95% confidence intervals.

To measure whether being a PE student influenced acceptable pain endurance scores, a mixed Factorial ANOVA with Field of Study as the independent variable and the five Activities and Task Severity (i.e., weak and strong) as the repeated measures variables was performed. Mauchly’s test revealed that the assumption of sphericity was violated for both Activity, *Χ*^2^ (9) = 700.57, *p* < .001, and the interaction between Activity and Task Severity, *Χ*^2^ (9) = 83.47, *p* < .001. For that reason, the Greenhouse-Geisser correction was applied to the degrees of freedom (for Activity, ε = .44; for the interaction between Activity and Task Severity, ε = .89). Participants scored acceptable contextual pain endurance more highly when Task Severity was strong (*M* = 6.35, *SD* = 2.03) compared to weak (*M* = 4.64, *SD* = 1.85; *F*(1, 299) = 871.83, *p* < .001, $\eta_{p}^{2}$ = .75). Main effects were also found for Field of Study, *F*(1, 299) = 14.50, *p* < .001, $\eta_{p}^{2}$ = .05, and Activity, *F*(1.76, 525.56) = 56.05, *p* < .001, $\eta_{p}^{2}$ = .16, with PE students more likely to accept higher pain in others than non-PE. A similar interaction effect of Field of Study on Activity was found, *F*(1.76, 525.56) = 21.77, *p* < .001, $\eta_{p}^{2}$ = .07. Acceptable contextual pain endurance scores did not significantly differ between PE and non-PE students regarding the academic and social activities. See Figure 2 for the mean scores and 95% confidence intervals of all activities displayed by Field of Study.

**Figure 3.**
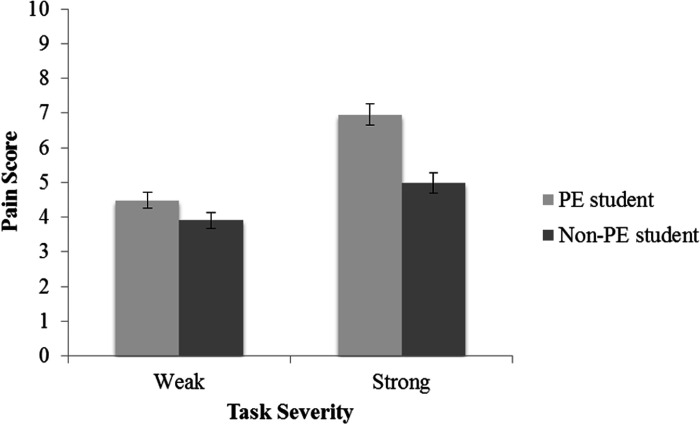
Mean scores for acceptable contextual pain endurance in weak and strong sports-related activities, divided by field of study. Error bars represent 95% confidence intervals.

An interaction effect between Field of Study and Task Severity confirmed the hypothesis that a higher immersion in sporting culture will lead to a stronger discrimination between low and high tasks severities in a sporting context (see Figure 3).

## Discussion

An extensive body of literature has highlighted the importance of socio-cultural factors in determining how an individual interprets and responds to, pain (Bendelow & Williams, 1995; Edwards et al., 2001; Wandner et al., 2012). One such example is the influence of sporting culture, which is often noted for its particular tolerance of physically risky behaviour (Curry & Strauss, 1994; R. T. Smith, 2008) and a willingness to accept pain that would likely be considered unacceptable in other contexts (Heil, 2012; Safai, 2003; Schneider et al., 2019). To date, most research in this area has focused on pain perception in the self (Forsythe et al., 2011; Nixon, 1994; Weinberg et al., 2013), with less being known about the effects immersion in sporting culture on attitudes towards pain in others outside of the clinical domain (Pike, 2005; Roderick et al., 2000). To address this question, the present study asked student participants to judge the level of pain that a peer should reasonably endure in a variety of contexts.

Findings demonstrated that all participants clearly discriminated between the relative “importance” of activities (measured in this study as task severity) when determining what is an acceptable pain level for a peer to endure. These attitudinal differences in acceptable pain tolerance between the strong and weak exemplars of each activity highlight the importance of contextual factors, and are conversant with the broad principles outlined by Bendelow (2006) and Garland (2012), regarding the socio-cultural character of individuals’ attitudes towards pain in general. Pertinently, however, high immersion in competitive sporting culture – as measured through field of study (and the 98.6% rate of weekly involvement in rigorous competitive sport endemic therein) – significantly enlarged the effect of task severity, but only with respect to the sport-related tasks. In the academic and social tasks, this was not the case.

These results appear incompatible with accounts that propose that individuals with high athletic identity simply have more natively competitive personalities than their less-sporting counterparts (Saragiotto et al., 2014), or at least any corollary proposal that such individuals would inherently impose highly competitive standards upon others evenly across sporting and non-sporting contexts. The inference herein does remain, however, that the prospective PE teachers involved in this study might well be inclined to push their students to persevere through higher levels of pain during sports and physical activity than would likely be acceptable to most; this being particularly so in contexts deemed of high importance.

With respect to the above, it is imperative to recognise that the line between “discomfort” and actual pain is far from self-evident, nor itself independent of individual context (Pageaux, 2016). In terms of robust physical activity, some level of discomfort – i.e. the everyday exertional perceptions associated with taxing the circulatory and respiratory organs, and with localised muscle fatigue – will likely be anticipated or even welcomed by exercisers themselves, as evidence of successful engagement. Such sensations, typically felt within the working muscles, are a common and entirely appropriate response to productive exercise in both adults and children (Kane et al., 2010; Robertson et al., 2009; S. A. Smith, 2014). It would be naïve, therefore, not to acknowledge that in some contexts, encouraging individuals to work through some degree of exercise-induced discomfort may well be a warranted activity; during training regimes designed to induce anaerobic, strength and power adaptations (Nemeth et al., 2005), for example.

It must be reiterated, however, that the experience of exercise-oriented pain is an alarm, warning the individual of actual or impending injury (Tesarz et al., 2012). A responsible agent such as a teacher must therefore be attentive to such distinctions, and be wary of making suppositions regarding what is likely “just” contextually-appropriate discomfort and what an individual might be experiencing as pain. There is no simple and externally-inferable line between the two (Pageaux, 2016). For a teacher, coach or trainer to – consciously or otherwise – expect his/her charges to *consistently* endure pain during sports and exercise can lead directly to avoidable injury and/or chronification of existing injury (Schneider et al., 2019). Moreover, it is important to recognise that it is exactly those under the care of PE teachers that might be most vulnerable to such acculturated attitudes, given evidence that children and adolescents often have limitations in their ability to fully recognise pain cues as a signal cease an activity, due to as-yet undeveloped experience and knowledge (Nemeth et al., 2005). This has implications not only for the health of the PE participant, but also well-established legal ramifications regarding duty of care for education and sport providers (DiCello, 2001; National Education Union, 2019).

It is important to register that this exploratory study has a number of limitations. Firstly, one should be mindful that espoused attitudes do not automatically *equate* to current or prospective behaviours, both of which have their own contexts, although there is little doubt that they exercise a degree of solid general governance (Holland et al., 2002; Link et al., 1999). Secondly, the use of a single-item (1-10) scale to assess attitudes towards pain in others could also be identified as a limitation of the study’s core method. Herein, such an approach was selected simply for its parity with the instruments typically used in clinical pain assessment (Jensen & Karoly, 2001). While some authors have argued that multiple-item measures are more likely to pragmatically capture an individual’s beliefs, perceptions and attitudes, this assessment is far from unanimous, particularly when the construct being measured is narrow in focus, unidimensional and unambiguous (Diamantopoulos et al., 2012; Gardner et al., 2016; Loo, 2002). Thirdly, it should be observed that a relatively high percentage (41.4%) of the non-PE students in this study were also regularly involved in competitive sporting activities. While not an uncommon rate of involvement among university students, in the UK at least, this is higher than that in the wider adult public (Sport England, 2019). It is therefore possible that the attitudinal differences between qualified physical educators and other *teachers* could actually be further magnified; this is a clear avenue for additional study. Finally, ethnicity-related differences were not addressed. This also might a be an instructive area for subsequent investigation, given the complex evidence relating to culture, ethnicity and personal pain attitudes (Cleland et al., 2005; Edwards & Fillingim, 2001; Forsythe et al., 2011; Wandner et al., 2012).

## Conclusions

It is contended that this exploratory research has evident import for the broader literature on pain, culture and context. Herein, high immersion in competitive sporting culture was demonstrated to significantly increase ratings of how much pain a peer should endure before abandoning particular sport-related activities. Although the approach adopted is not without inferential limitations, as noted, the nature of the findings, and their close correspondence with those in extant literature on cultural attitudes towards personal pain, suggests that it may prove a useful point-of-departure for future studies addressing acculturated attitudes towards pain in others. Above all, however, given the particular focus upon physical education and educators, this study provides a preliminary quantitative insight into how “risky” attitudes towards pain might be perpetuated within the grassroots culture of sport from one generation to the next. From a health psychology perspective this further illuminates a well-documented problem in the culture of sport, and also strengthens the case for more active intervention in tertiary education to help arrest its day-to-day reproduction.

## Note

1 Findings from which are themselves anticipated as the topic of a forthcoming qualitative paper.
